# The Effect of Sex and Wealth on Population Attributable Risk Factors for Dementia in South Africa

**DOI:** 10.3389/fneur.2021.766705

**Published:** 2021-11-25

**Authors:** Kirsten Bobrow, Tina Hoang, Deborah E. Barnes, Raquel C. Gardner, Isabel E. Allen, Kristine Yaffe

**Affiliations:** ^1^Global Brain Health Initiative at UCSF, University of California, San Francisco, San Francisco, CA, United States; ^2^Department of Psychiatry and Behavioral Sciences, University of California, San Francisco, San Francisco, CA, United States; ^3^Northern California Institute for Research and Education, San Francisco, San Francisco, CA, United States; ^4^Department of Epidemiology and Biostatistics, University of California, San Francisco, San Francisco, CA, United States; ^5^San Francisco Veterans Affairs Health Care System, San Francisco, San Francisco, CA, United States; ^6^Department of Neurology, University of California, San Francisco, San Francisco, CA, United States

**Keywords:** dementia, risk factor (RF), modifiable, Africa, sex, wealth, population attributable risk (PAR)

## Abstract

**Background and Aims:** South Africa is a middle-income country with high levels of income inequality and a rapidly aging population and increasing dementia prevalence. Little is known about which risk factors for dementia are important and how they differ by social determinants of health as well as key demographic characteristics such as sex and wealth. We sought to calculate the population attributable risks (PARs) for established potentially modifiable risk factors for dementia among these different groups.

**Methods:** We obtained risk factor prevalence from population-based surveys for established dementia risk factors (diabetes, midlife hypertension, midlife obesity, physical inactivity, depression, smoking, low educational attainment, social isolation). We used relative risk estimates reported in previous meta-analyses and estimated PARs using Levin's formula and accounting for communality. We tested for one-way and two-way interactions by sex and wealth using Pearson's χ2. In stratified analyses, we performed tests for trend using logistic regression.

**Results:** The prevalence of established risk factors for dementia ranged from 5% for depression to 64% for low education. After accounting for communality, the risk factors contributing the greatest PAR were low education (weighted PAR 12%, 95% CI 7% to 18%), physical inactivity (9, 5–14%), and midlife hypertension (6, 5–14%). Together, 45% of dementia cases may be attributable to modifiable risk factors (95% CI 25–59%). We found significant interactions (*p* < 0.005) between sex, wealth, or both (sex ^*^ wealth) and each risk factor except social isolation and physical activity. Low education was inversely associated with wealth in both male and female. The PAR for midlife hypertension, obesity, and diabetes was associated with increasing wealth, and was higher in female. In contrast, the PAR for smoking was higher in male (8% vs. 2%) and was associated with increasing wealth among female only. We found that either a strategy of large reductions in selected risk factors with the highest PAR (midlife hypertension, smoking, physical inactivity) or small reductions across all risk factors could potentially reduce dementia cases by as many as 250,000 by 2050.

**Discussions:** The potential impact on dementia risk by decreasing exposure to established dementia risk factors is large and differs by sex and social determinants of health like wealth. Risk factor PAR should inform national and local health policy dementia initiatives in South Africa including which risk factors to target in the whole population and which to target in high-risk groups for maximum public health benefit.

## Background

Worldwide, the number of people living with dementia is increasing (1). The biggest increase will be in low- and middle-income countries (LMICs) with large populations and life expectancy that is rising faster than in high-income countries (HICs) (2, 3). Results from cohort studies in some HICs suggest that the incidence of dementia has decreased over the past one or two decades suggesting it may be preventable on a public health level (4–7). Many studies, primarily among HICs, have established that midlife hypertension and obesity, diabetes, low education, physical inactivity, smoking, and depression are important potentially modifiable risk factors for dementia (8–10).

The population attributable risk (PAR) estimates are useful measures to translate the public health importance of research findings by providing estimates of the potentially preventable risk factors on downstream outcomes (11). The PAR estimates for established dementia risk factors have been published primarily for high and for a few middle- and low-income settings and are being used to frame discussions on policies and other interventions to prevent or delay dementia onset (10, 12, 13). Yet, little is known about the important dementia risk factors and PARs in countries from Africa (14–17). Furthermore, dementia prevalence is known to vary by sex and income, and other social determinants of health; yet little is known about how such variation should be incorporated into public health response strategies at national and subnational levels (18).

South Africa is a MIC with high levels of income inequality as well as a rapidly aging population and increasing dementia prevalence (19). The population is racially diverse and highly urbanized (67% of South Africans live in cities). We report on the contribution of the established risk factors to the potentially preventable burden of dementia in South Africa and how these vary by sex, wealth, and residence.

The health system already manages a quadruple disease burden (infectious disease, non-communicable diseases [NCDs], maternal/child morbidity and mortality, and trauma) and it is unclear how best to incorporate dementia prevention strategies into the services (20). We report on the potential number of dementia cases that could be averted using targets laid out in the WHO “25 by 25” campaign to decrease mortality by cardiovascular disease by 25% by 2025 (as part of the Global Action Plan for the Prevention and Control of NCDs 2013–2020) compared to a 10% decrease per decade in prevalence across all risk factors (21, 22).

## Methods

We used previously published definitions for seven important potentially modifiable dementia risk factors: midlife hypertension and obesity, diabetes, tobacco smoking, physical inactivity, educational attainment, and depression (8, 9, 23, 24). For midlife hypertension and obesity, we replicated previously published methods to calculate midlife risk factor prevalence estimates by calculating the percentage of the population that had the risk factor and was in the age group of interest (35–65 years) (8). Supplementary Table 1 lists the definitions of each risk factor included in the analysis.

We calculated prevalence estimates for each of the seven risk factors using data from the 2016 South African Demographic Health and Surveillance study (SADHS 2016). The SADHS 2016 is a nationally representative household survey of a wide range of health, population, and nutrition indicators that uses a stratified sampling design. We used data from 10,336 adults [mean age 39 years, Interquartile Range (IQR) 24–51], who completed the health module including medical history, anthropometry, blood pressure measurement, and a blood draw (25). We calculated prevalence estimates using these data for low education, midlife hypertension and obesity, diabetes, and tobacco smoking. We compared low-education prevalence by 10-year age strata because educational attainment of older adults in South Africa is impacted by discriminatory Apartheid laws and legislation including the Bantu Education Act (1953), which limited access to education for black South Africans until the early 1990s (26).

For risk factors not assessed by the SADHS 2016, physical activity, depression, and social isolation, we calculated prevalence and communality using South Africa (wave 1) data from the WHO Study on global AGEing and adult health (SA-SAGE), a nationally representative survey of adults aged 50 years (mean age 52 years, IQR 44–58) and older sampled in 2007–2008 (27).

Wealth tertiles were calculated using the raw index of household wealth calculated for each survey. Household wealth index is an established methodology to estimate income and consumption in low-income settings and DHS surveys (28). Briefly, wealth indices are calculated based on the number and kinds of consumer goods the household owned, ranging from a television or car to the number and type of animal stock owned to housing characteristics, such as the source of drinking water, toilet facilities, and flooring material (29). A recent review of the correlation between wealth index and income and consumption shows a moderate correlation between these indices (30). Both surveys defined residence (urban/rural) during the sampling process using enumeration areas from either 2011 or 2001 census data, respectively.

We used relative risk estimates and their 95% CIs from the most recently available meta-analysis (8–10, 17). To account for the non-independence of individual risk factors and incomplete control of potential confounders in studies calculating relative risk estimates, we used similar methods to previous studies to calculate a communality weight (1 minus the proportion of the variance shared with other risk factors) (9). Briefly, we used data from adults over 65 years of age on the nine risk factors from the SA-SAGE survey (10). We performed a principal component analysis of the inter-risk factor tetrachoric correlation matrix. We used Kaiser criteria (eigenvalues ≥1) to select the number of principal components to extract (31). This analysis showed that our components explained 51% of the total variance among the nine risk factors (9). We calculated the sum of the square of all factor loadings to obtain the communality (13).

## Statistical Analysis

We used Levin's formula to calculate the PAR for each risk factor:


$$\begin{array}{l} PAR={\text{P}}_{\text{exp}}\times \left(RR-1\right)/1+{\text{P}}_{\text{exp}}\times \left(RR-1\right) \end{array}$$

*P*_exp_ is the prevalence of the exposure and RR the relative risk of disease due to that exposure (32). The PAR for a risk factor is the amount that the risk factor contributes to the outcome variance. We also calculated a combined PAR accounting for communality (8, 9).

To explore for differences in risk factors due to social determinants of health, we first tested for interactions by sex, wealth, and residence using Pearson's χ^2^. Tests for statistical significance were set at *p* < 0.005. We found residence (urban/rural) was collinear with wealth tertile and we excluded residence from further analyses. We then tested each risk factor for interactions by sex, wealth, and both (sex ^*^ wealth). We examined risk factor prevalence by sex and wealth index (tertile) strata and tested for trends using logistic regression models. We conducted sensitivity analyses using the lowest and highest prevalence estimates to see how these affect the PAR.

The total number of dementia cases was estimated by multiplying the total number of persons aged 65 years old or above (numbers available in https://data.worldbank.org) by the most recent dementia prevalence in South Africa (11%) (19). The number of dementia cases attributable to each risk factor was then estimated by multiplying the PAR estimates by the number of dementia cases. We next calculated the number of dementia cases potentially averted by a 10% decrease per decade in prevalence across all risk factors (8). We compared this to the number of cases potentially averted by focusing on the WHO “25 by 25” goals for non-communicable diseases as applied to preventing dementia namely, a 25% relative reduction in the prevalence of hypertension, 30% relative reduction in the prevalence of current tobacco smoking, and a 10% relative reduction in insufficient physical activity prevalence, and no increase in the prevalence of obesity or diabetes mellitus (21).

## Results

Table 1 shows the prevalence, relative risk, and communality, with the PAR adjusted for communality of each included risk factor, and an overall weighted PAR. Our results show that 45% (27–67%) of dementia cases in South Africa are due to exposure to the established risk factors. The risk factors contributing the greatest PAR were low education (weighted PAR 12%, CI 7–18%), physical inactivity (9, 5–14%), and midlife hypertension (6, 2–11%). Adjusting for communality decreased the size of individual PAR estimates slightly but did not change their rank.

**Table 1:** Risk factor prevalence in South Africa, relative risk associated with dementia, communality, Population Attributable Risk (PAR), and weighted PAR.

**Risk factor**	**Prevalence**	**Relative risk**	**Communality**	**PAR 95% CI)**	**Weighted PAR (95% CI)**
Low education	64%	1.6 (1.3–2.0)	50%	28% (16–39)	12% (7–18)
Midlife hypertension	28%	1.6 (1.2–2.2)	74%	14% (5–25)	6% (2–11)
Midlife obesity	18%	1.6 (1.3–1.9)	44%	10% (5–14)	4% (2–6)
Diabetes mellitus	12%	1.5 (1.3–1.8)	24%	5% (3–8)	2% (2–4)
Smoking	20%	1.6 (1.2–2.2)	39%	11% (4–43)	5% (2–20)
Depression	5%	1.9 (1.6–2.3)	28%	5% (3–6)	2% (1–3)
Physical inactivity	62%	1.4 (1.2–1.7)	30%	20% (11–30)	9% (5–14)
Social isolation	14%	1.6 (1.3–1.9)	23%	8% (4–11)	4% (2–5)
**Overall weighted PAR**				**45% (27–67)**	

The PAR for low education differed by wealth strata but not sex (*p* for interaction with sex = 0.1880, *p* for interaction with wealth <0.0000). For midlife hypertension, diabetes, obesity, and smoking, the PAR estimates differed by sex and wealth (*p* for sex ^*^ wealth interaction <0.005). For depression, the estimates differed by sex only (higher in female). We did not find interactions by sex or wealth for social isolation or physical activity. Individually weighted PAR estimates by sex and wealth are shown in Supplementary Tables 3, 4.

**Figure 1:**
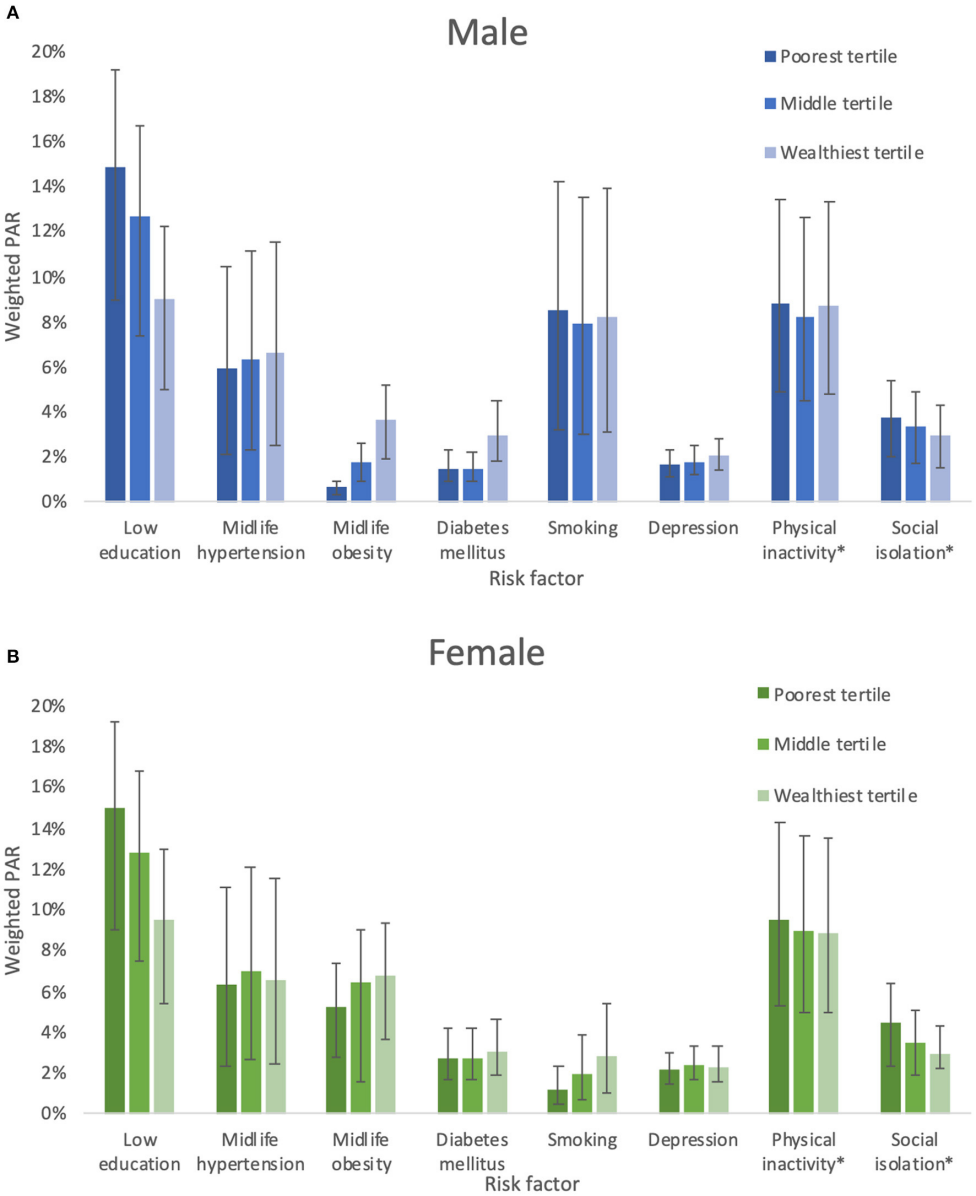
**(A)** Weighted PAR for dementia risk factors in males. **(B)** Weighted PAR for dementia risk factors in females. *No significant interaction between sex or wealth and physical acitvity or social isolation.

Figures 1A,B show weighted PAR estimates for male and female by wealth tertile. The PAR for low education was associated with decreasing wealth in both male and female (*p* for interaction with sex = 0.1880, *p* for interaction with wealth <0.000; *p* for trend by wealth tertile <0.00). The prevalence of low education was associated with increasing age. The prevalence was lowest among adults aged 20–30 years (52, 49–55%), and increased with each decade to 81% (CI 77–85%) in people over the age of 60 years (*p* for trend <0.000).

Midlife obesity PAR was associated with increasing wealth in both male and female (*p* for interaction with sex and wealth <0.000; *p* for trend by wealth tertile <0.000), although the PAR for female was several times higher (e.g., 1% in male in the lowest wealth tertile compared with 5% in female). The results for midlife obesity were similar. The PAR for smoking was 8% (4–11%) in male compared with 2% (1–3%) in female. In female, but not male, the PAR for smoking was associated with increasing wealth (male *p* = 0.45, female *p* < 0.001).

**Figure 2:**
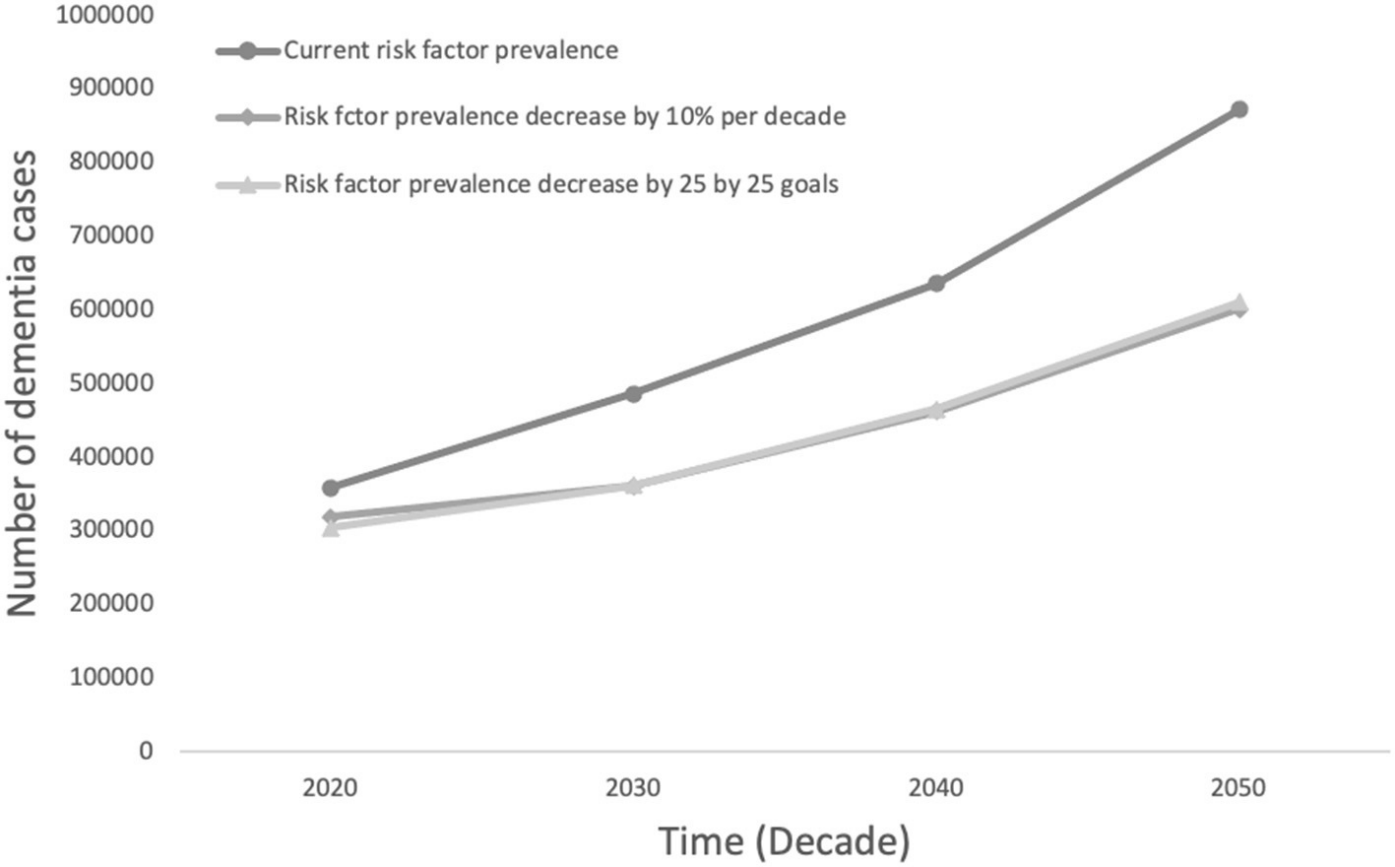
Projected number of dementia cases in South Africa by decrease in risk factor prevalence comparing the potential impact of a 10% decrease in all risk factors to a decrease in line with 25 by 25 goals.

Figure 2 shows the projected number of dementia cases in South Africa using current prevalence estimates and comparing two strategies to decrease relative risk factor prevalence by 10% for each risk factor or focused on reducing the prevalence of hypertension by 25%, smoking by 30%, and physical activity by 10% over the next three decades. The reduction in numbers of people with dementia are similar by strategy; small reductions across all risk factors could potentially prevent 273, 440 cases, compared to 262, 387 cases focusing on selected 25 by 25 targets.

## Discussions

The potentially modifiable risk factors for dementia are common in South Africa and may account for 45% of dementia suggesting that reduction of these could have large effects on future dementia incidence and prevalence. We also found important variation in risk factor prevalence within the country by wealth and sex strata. We found that large reductions in a few key factors (midlife hypertension, smoking, physical inactivity) would prevent about the same number of cases as a broader strategy focusing on decreasing each risk factor by a little.

Researches from high-income settings including the United States of America, United Kingdom, and Europe have shown that between a half and a third of dementia cases may be preventable (8–10). Yet, very few studies have been published from countries in Africa on the preventable burden of dementia. We are aware of only one similar study from Portugal, Brazil, and Mozambique, which found that the potentially preventable proportion of dementia cases due to the established risk factors was much lower in Mozambique and Brazil than in Portugal (about 24 and 32% compared to 40%) (33). South Africa is a MIC with high levels of inequality similar to Brazil. Our finding of a larger proportion of potentially preventable dementia is due to the higher prevalence of many risk factors in South Africa as compared to Brazil (for example, midlife hypertension in South Africa is 28% compared to 7.4% reported in Olivera et al.).

Our results are similar to a recent analysis of 10/66 data from eight LMICs, not including any countries from Africa, which showed that PAR estimates for these risk factors were generally higher than in HICs (between 37 and 57%) because of higher risk factor prevalence and that the contribution of individual risk factors varied across countries (13).

Our analyses build on and extend findings in this area by exploring the influence of key social determinants of health including sex, urban or rural residence, and wealth tertiles on risk factor prevalence and therefore PAR. Such findings may be useful to policymakers to understand the impacts of broad dementia prevention interventions compared to targeting specific risk factors and for understanding with whom to target interventions (for example tobacco cessation among wealthier females). South Africa has a two-tiered health system, with over 80% of the population accessing all of their care through public sector facilities, which allow for broad reach of health policies (34).

Comparing prevalence estimates from the DHS Survey in 1998, our findings also show social changes since the end of Apartheid have resulted in dramatic shifts in modifiable risk factors. For example, access to education has improved (in 1998, 45% of females and 38% of males aged 65 years or older reported having received no education compared with 34% of female and 24% of males in 2016); however, obesity and diabetes are increasingly prevalent, and tobacco use in female is increasing (25, 35). These will impact the risk and incidence of dementia in the future.

Our study has several important strengths: we used recent population-based prevalence surveys from South Africa using measured blood pressure, anthropometry, HbA1c, and standardized algorithms to calculate prevalence estimates and to calculate communality scores. We also used the best available relative risk estimates from recent systematic reviews and meta-analyses.

However, there are several limitations. As far as possible, we used previously published definitions for risk factors; however, the prevalence of some risk factors, such as health-care worker diagnosis of depression, may be under or overestimated based on health-seeking behavior. We used published relative risk estimates, which are primarily from studies in high-income settings. However, we think the causal mechanisms are similar (for example, smoking leading to cerebrovascular disease and vascular dementia), and large meta-analyses provide more precise risk estimates. The PAR estimates assume that the association between the risk factor and the outcome is causal and that the size of the relative risk is a reasonable estimate of the impact on disease incidence of removing a risk factor. It is unlikely that risk factors will be completely eliminated; however, a reduction in risk factors may delay the onset of dementia and thereby reduce case numbers. We used wealth index as a proxy measure of income to estimate socioeconomic status, although this a well-established indictor used in global health to understand health inequalities and to control for potential confounding; it has limitations including that it is a measure of long-term household assets (it does not reflect short to medium-term changes). Other limitations include recall error, differential exclusion of expenses, changes in asset costs (e.g., new cheaper imports from Asia), currency exchange rates, choice of deflator, and method for calculating the index (simple weighted average vs. principal component analysis). The data on physical activity, depression, and social isolation are from Wave 1 of the SA-SAGE in 2007–8, while the remaining risk factors are from the most recent SADHS in 2016.

Dementia is a clinical syndrome due to a variety of underlying pathophysiological processes. The effects of entirely removing a single risk factor may not be easy to ascertain, especially, if other risk factors become more prevalent. Also, many of the established risk factors cluster together (for example, smoking, hypertension, diabetes, depression, and physical inactivity) and interact with each other (for example, smoking cessation and increase in weight gain) in ways that suggest the need for a holistic approach to risk management (36). Finally, there are other potentially modifiable risk factors that were not included in our estimates and that may be important locally, such as traumatic brain injury, HIV, and alcohol (20).

Our results showing the portion of cases that could be prevented comparing a global reduction in risk factor prevalence of 10% to a reduction in line with the 25 by 25 goals is important in South Africa. Demographically, the country is young (median age 27 years) and, while it is rapidly aging, the absolute number of people over 65 years is still small (5.4 million). The infrastructure to support research and interventions to prevent dementia is still developing. In contrast research, health, and policy infrastructure to address common NCDs is already well developed. Leveraging existing NCD research and policy initiatives is an opportunity to expand dementia research using existing platforms (low cost, high yield) and the reach of dementia research findings (for example, initiatives to reduce hypertension prevalence may have longstanding positive impacts across a variety of health outcomes). In addition, results from our and other studies show smoking remains prevalent and is increasing among some groups (e.g., young wealthy female) (37). Tobacco cessation support (non-pharmacological and pharmacological) is important but relatively neglected in South Africa and other LMICs (38). Finally, access to education in South Africa has increased substantially but educational achievement quality is poor in comparison to international metrics (39); according to the World Bank, a child who starts school at age 4 will receive 10.2 years of basic education but adjusting for what children actually learn, expected years of school are only 5.6 years (40). This coupled with limited access to higher education and high unemployment (i.e., limited opportunity for other cognitive stimulation and skills acquisition) likely mean the long-term cognitive benefits of education will not be realized until the education system is significantly improved.

Very little is known about the established and emerging risk and protective factors affecting cognitive function, brain aging, and dementia risk in most countries in Africa. Our study has shown that the established risk factors for dementia are common in South Africa and may account for a sizeable burden of potentially preventable dementia cases. The existing policy initiatives to reduce the burden of common NCDs could reduce the impact of dementia in South Africa in the coming decades and should be supported and strengthened.

## Data Availability Statement

Publicly available datasets were analyzed in this study. This data can be found at: South Africa DHS 2016: https://dhsprogram.com/methodology/survey/survey-display-390.cfm and WHO SAGE South Africa Wave 1: https://apps.who.int/healthinfo/systems/surveydata/index.php/catalog/5.

## Ethics Statement

Ethical review and approval was not required for the study on human participants in accordance with the local legislation and institutional requirements. The patients/participants provided their written informed consent to participate in this study.

## Author Contributions

KB, KY, and RG: conception and design of the study. KB: acquisition of and analysis of data. KB, KY, RG, IA, and DB: analysis and interpretation of data. KB and KY: drafting manuscript. KB, KY, TH, RG, IA, and DB: revising manuscript for important intellectual content. KB, KY, TH, RG, IA, and DB approval of the version of the manuscript submitted. All authors contributed to the article and approved the submitted version.

## Funding

This work was supported through a 2019 Pilot Awards for Global Brain Health Leaders by the Global Brain Health Institute (GBHI), and Alzheimer's Association, Alzheimer's Society. Grant Number GBHI ALZ UK-20-642357.

## Conflict of Interest

The authors declare that the research was conducted in the absence of any commercial or financial relationships that could be construed as a potential conflict of interest.

## Publisher's Note

All claims expressed in this article are solely those of the authors and do not necessarily represent those of their affiliated organizations, or those of the publisher, the editors and the reviewers. Any product that may be evaluated in this article, or claim that may be made by its manufacturer, is not guaranteed or endorsed by the publisher.
