# Supplementary material for: The Effect of Sex and Wealth on Population Attributable Risk Factors for Dementia in South Africa
Source: Front Neurol. 2021 Nov 25;12:766705. doi: 10.3389/fneur.2021.766705 (PMC8655099; doi:10.3389/fneur.2021.766705)
Supplement: Supplementary file 1 [file Data_Sheet_1.docx]

Supplemental table 1. Definitions of risk factors used in analyses

**Low educational attainment**

The proportion of adults form the SADHS 2016 survey with a self-reported International Standard Classification of Education level of 2 or less (pre-primary, primary, and lower secondary education). (8,20)

**Midlife hypertension**

Hypertension was defined using SADHS 2016 data. Respondents were classified as having hypertension if they had a systolic blood pressure >140 mmHg or diastolic blood pressure >90 mmHg calculated using the mean of two seated measurements or were currently taking anti-hypertensive medication. Age stratified prevalence was used in conjunction with national census data to calculate the proportion of the adult population (age >=18 years) that had hypertension and were between the ages of 35 years and 64 years. (8,20)

**Midlife obesity**

Obesity was defined as a body-mass index > 30 kg/m² using measured height and weight data from the SADHS 2016 survey. Age stratified prevalence was used in conjunction with national census data to calculate the proportion of the adult population (age>=18 years) that was obese and between the ages of 35 years and 64 years. (8,20)

**Diabetes mellitus**

Glycated hemoglobin (HbA1c) was measured in SADHS 2016 using dried blood spot specimens. Standard HbA1c measurement is usually based on blood samples rather than DBS specimens. To account for this difference in specimen type, a calibration factor was applied (venous = (DBS-0.228)/0.9866.) Diabetes mellitus was defined as an HbA1c >= 6.5% (International Expert Committee 2009). (20) We calculated the prevalence of diabetes among adults aged 20 to 79 years old. (8,20)

**Smoking**

Tobacco smoking was defined as self-reported current daily cigarette smoking using SADHS 2016 survey data. (20)

**Depression**

Diagnosis of major depressive episode was calculated from SA-SAGE survey data using an algorithm that accounted for reporting symptoms of depression during the past 12 months. (20,22,23) The detailed symptom questions and the algorithm are provided in the supplementary material (Table 5).

**Physical inactivity**

The prevalence of physical inactivity was defined as proportion of adults who do not do either 20 min of vigorous activity on 3 or more days or 30 min of moderate activity on 5 or more days per week. Physical activity was measured in the SA-SAGE survey using the Global Physical Activity Questionnaire (GPAQ), which collected information about physical activity in three domains (activity at work (paid/unpaid), travel to and from places, recreational activities) and sedentary behavior. (21)

**Social isolation**

Social isolation was defined as having over or going to visit friends or relatives fewer than once or twice per month and going out fewer than once or twice per month using data from the SA-SAGE survey.

Supplementary table 2. Low education by age group

| 10 – year Age groups | Prevalence Low Education |
| --- | --- |
| <20 yrs | 76% (70% - 81%) |
| 20 to 30 yrs | 52% (49% to 55%) |
| 30 to 40 yrs | 58% (54% - 62%) |
| 40 to 50 yrs | 61% (57% - 65%) |
| 50 to 60 yrs | 75% (71% - 79%) |
| 60 + yrs | 81% (77% - 85%) |

Supplementary table 3. Individually weighted PAR for risk factors by sex

| SEx (95% CI) | Male | | Female | |
| --- | --- | --- | --- | --- |
| Low education | 12.3% | (7.2%-16.3%) | 12.5% | (7.3%-16.5%) |
| Midlife hypertension | 6.3% | (2.3%-11.0%) | 6.6% | (2.5%-11.6%) |
| Midlife obesity | 2.0% | (1.0%-3.0%) | 6.1% | (3.3%-8.6%) |
| Diabetes mellitus | 1.9% | (1.2%-3.0%) | 2.8% | (1.7%-4.3%) |
| Smoking | 8.2% | (3.1%-13.9%) | 2.0% | (0.7%-3.8%) |
| Depression | 1.8% | (1.2%-2.5%) | 2.2% | (1.5%-3.1%) |
| Physical inactivity | 8.7% | (4.8%-13.3%) | 9.1% | (5.1%-13.8%) |
| Social isolation | 3.3% | (1.7%-4.8%) | 3.8% | (2.0%-5.4%) |

Supplementary table 4. Individually weighted PAR for risk factors by wealth tertile

| Wealth Index (95%CI) | Poorest tertile | | Middle tertile | | Wealthiest tertile | |
| --- | --- | --- | --- | --- | --- | --- |
| Low education | 14.9% | (8.9%-19.2%) | 12.8% | (7.5%-16.8%) | 9.3% | (5.2%-12.6%) |
| Midlife hypertension | 6.1% | (3.3%-8.6%) | 6.7% | (3.6%-9.3%) | 6.6% | (3.6%-9.2%) |
| Midlife obesity | 3.1% | (1.6%-4.5%) | 4.3% | (2.3%-6.2%) | 5.3% | (2.8%-7.5%) |
| Diabetes mellitus | 2.3% | (1.4%-3.9%) | 2.2% | (1.4%-3.8%) | 3.0% | (1.8%-5.1%) |
| Smoking | 4.5% | (2.4%-6.4%) | 4.7% | (2.5%-6.6%) | 5.2% | (2.8%-7.4%) |
| Depression | 1.9% | (0.6%-1.9%) | 2.2% | (0.7%-2.2%) | 2.2% | (0.7%-2.2%) |
| Physical inactivity | 9.2% | (7.3%-16.5%) | 8.7% | (6.9%-15.8%) | 8.8% | (6.9%-15.9%) |
| Social isolation | 4.2% | (2.2%-6.0%) | 3.5% | (1.8%-5.0%) | 3.0% | (1.5%-4.3%) |

Supplementary table 5.

Questions asked in the SA-SAGE survey and the algorithm to define depression (20,22,23)

| **Depression** | 1 | During the last 12 months, have you had a period lasting several days when you felt sad, empty or depressed? |
| --- | --- | --- |
|  | 2 | During the last 12 months, have you had a period lasting several days when you lost interest in most things you usually enjoy such as personal relationships, work or hobbies/recreation? |
|  | 3 | During the last 12 months, have you had a period lasting several days when you have been feeling your energy decreased or that you are tired all the time? |
|  |  | If any of the above three questions are yes then following set of questions were asked |
|  | 4 | Was this period [of sadness/loss of interest/low energy] for more than 2 weeks? |
|  | 5 | Was this period [of sadness/loss of interest/low energy] most of the day, nearly every day? |
|  | 6 | During this period, did you lose your appetite? |
|  | 7 | Did you notice any slowing down in your thinking? |
|  | 8 | Did you notice any problems falling asleep? |
|  | 9 | Did you notice any problems waking up too early? |
|  | 10 | During this period, did you have any difficulties concentrating; for example, listening to others, working, watching TV, listening to the radio? |
|  | 11 | Did you notice any slowing down in your moving around? |
|  | 12 | During this period, did you feel anxious and worried most days? |
|  | 13 | During this period, were you so restless or jittery nearly every day that you paced up and down and couldn’t sit still? |
|  | 14 | During this period, did you feel negative about yourself or like you had lost confidence? |
|  | 15 | Did you frequently feel hopeless - that there was no way to improve things? |
|  | 16 | During this period, did your interest in sex decrease? |
|  | 17 | Did you think of death, or wish you were dead? |
|  | 18 | During this period, did you ever try to end your life? |
|  | **Algorithm** | **To ascertain the depression from this set of questions two set of variables were computed. First set was based on the questions1, 2, 3, 4, 5 and 16. From this set three variables were computed taking values 0 and 1: a) first variable takes value 1 if response to any of questions 1, 4, and 5 was yes. b) second variable takes value 1 if question 2 or 16 has response yes. c) the third variable takes value 1 if question 3 has response yes. The second set of variables was based on questions 6, 7, 8, 9, 10, 11, 12, 13, 14, 15, 17 and 18. From these questions seven variables were computed. a)first variable takes value 1 if response to questions 14 or 15 is yes. b) second variable takes value 1 if response to questions 12 or 13 is yes. c) third variable takes value 1 if questions 17 or 18 has response yes. d) fourth variable takes value 1 if questions 7 or 10 has response yes. e) fifth variable takes value 1 if response to questions 11 is yes. f) sixth variable takes value 1 if response to questions 8 or 9 is yes. g) seventh variable takes value 1 if the response to question6 is yes. These newly created variable from the respective sets were added to obtain two new variables : first consisting sum of first set of variables (maximum value 3) and second consisting sum of second set of variables (maximum value 7). Based on these two variables, a respondent is said to suffer from depression if he has value for the first variable to be 2-plus and the value for second variable to be 4-plus.** |
